# Solvothermal synthesis of n-type Bi_2_(Se_*x*_Te_1−*x*_)_3_ nanoplates for high-performance thermoelectric thin films on flexible substrates

**DOI:** 10.1038/s41598-020-63374-0

**Published:** 2020-04-14

**Authors:** Yuki Kimura, Ryotaro Mori, Susumu Yonezawa, Hayato Yabuki, Hiromasa Namiki, Yuichi Ota, Masayuki Takashiri

**Affiliations:** 1 Department of Materials Science, Tokai University, 4-1-1 Kitakaname, Hiratsuka, Kanagawa 259-1292 Japan; 2 Tokyo Metropolitan Industrial Technology Research Institute, 2-4-10, Aomi, Koto-ku, Tokyo, 135-0064 Japan

**Keywords:** Materials science, Nanoscale materials, Nanoparticles

## Abstract

To improve thermoelectric performance of materials, the utilization of low-dimensional materials with a multi-alloy system is a promising approach. We report on the enhanced thermoelectric properties of n-type Bi_2_(Se_*x*_Te_1−*x*_)_3_ nanoplates using solvothermal synthesis by tuning the composition of selenium (Se). Variation of the Se composition within nanoplates is demonstrated using X-ray diffraction and electron probe microanalysis. The calculated lattice parameters closely followed Vegard’s law. However, when the Se composition was extremely high, an impurity phase was observed. At a reduced Se composition, regular-hexagonal-shaped nanoplates with a size of approximately 500 nm were produced. When the Se composition was increased, the shape distribution became random with sizes more than 5 μm. To measure the thermoelectric properties, nanoplate thin films (NPTs) were formed on a flexible substrate using drop-casting, followed by thermal annealing. The resulting NPTs sufficiently adhered to the substrate during the bending condition. The electrical conductivity of the NPTs increased with an increase in the Se composition, but it rapidly decreased at an extremely high Se composition because of the presence of the impurity phase. As a result, the Bi_2_(Se_*x*_Te_1−*x*_)_3_ NPTs exhibited the highest power factor of 4.1 μW/(cm∙K^2^) at a Se composition of *x* = 0.75. Therefore, it was demonstrated that the thermoelectric performance of Bi_2_(Se_*x*_Te_1−*x*_)_3_ nanoplates can be improved by tuning the Se composition.

## Introduction

With the development of information technology including the Internet of things (IoT), the importance of thermoelectric materials that form flexible thin films has increased. This is because thermoelectric materials can directly convert thermal energy to electrical energy and vice versa. The electrical energy generated from the thermal energy via the Seebeck effect can be used to power wireless and wearable sensors^1–4^. The thermal energy produced from electrical energy via the Peltier effect can be used to cool electronic devices^5–7^. In both cases, the energy conversion efficiency depends on the dimensionless figure-of-merit *ZT*, defined as *ZT* = *S*^2^*σT*/*κ*, where *S* is the Seebeck coefficient, *σ* is the electrical conductivity, *κ* is the thermal conductivity, and *T* is the absolute temperature. In addition, the power factor *PF* defined by *PF* = *S*^2^*σ* is an important material property.

Among thermoelectric materials, bismuth telluride-based alloys which were developed in the 1950s are the most suitable materials for application to IoT technology^8–10^. This is because they exhibit the highest thermoelectric properties near 300 K. At this temperature, thermoelectric materials can be used as both thermoelectric generators and Peltier coolers. In particular, ternary alloys such as Bi_2_(Se_*x*_Te_1-*x*_)_3_ are known to exhibit superior performance compared to binary alloys such as Bi_2_Te_3_ and Bi_2_Se_3_^11–14^. Bismuth telluride-based alloys including binary and ternary alloys commonly have rhombohedral tetradymite-type crystal structures with the space group ${D}_{3d}^{5}(R\mathop{3}\limits^{-}m)$ and hexagonal unit cells^15^. The crystal structures of Bi_2_Te_3_ and Bi_2_Se_3_ binary and Bi_2_(Se_*x*_Te_1-*x*_)_3_ ternary alloys are presented in the supplemental information provided in Fig. S1. In the Bi_2_Te_3_ structure, the unit cell is composed of five covalently bonded monatomic sheets along the *c-*axis in the sequence –Te^(1)^–Bi–Te^(2)^–Bi–Te^(1)^–. The superscripts (1) and (2) denote the two different chemical states of the anions. The bonds between Te^(1)^ and Bi include both covalent and ionic bonds, while Te^(2)^ and Bi are covalently bonded. Moreover, a very weak van der Waals attraction exists between neighboring Te^(1)^ layers. In the Bi_2_Se_3_ structure, only Te atoms are replaced by Se atoms, and the structure of the unit cell is the same as that of the Bi_2_Te_3_. In the ternary alloys, Bi_2_(Se_*x*_Te_1-*x*_)_3_ is formed via the preferential replacement of Se atoms with Te^(2)^ atoms. When the Se atoms fully occupied the Te^(2)^ sites, they begin to fill the Te^(1)^ sites.

To further enhance the thermoelectric properties, an effective approach is to reduce the dimension of the materials. Hicks *et al*. theoretically predicated that materials with low dimensions exhibit improved thermoelectric properties due to the increased power factor caused by quantum confinement^16^. However, the difficulty of device fabrication and the fabrication cost increase as the dimension is significantly reduced. Considering these factors, 2D materials including layered structure and nanoplates have recently attracted broad attention^17–20^. In particular, nanoplates are highly suitable because they can be used to fabricate high-quality single crystals via a low-cost wet process^21–26^. As such, nanoplates made of bismuth telluride-based ternary alloys are promising thermoelectric materials. Soni *et al*. and Liu *et al*. prepared Bi_2_Te_3-*x*_Se_*x*_ nanoplates by varying the atomic composition using a polyol process^27,28^. Hong *et al*. prepared similar nanoplates using a microwave-assisted solvothermal method^29^. The bulk sintered samples were fabricated from Bi_2_(Se_*x*_Te_1-*x*_)_3_ nanoplates and exhibited excellent thermoelectric performances. These pioneering studies partially motivated our own investigation into Bi_2_Te_3−*x*_Se_*x*_ nanoplates with optimal atomic composition to form flexible thin films for application toward IoT technology. In addition, these studies motivated us to investigate the thermoelectric properties of nanoplates while maintaining their shape. In our previous reports, we fabricated binary alloy nanoplates such as Bi_2_Te_3_ and Sb_2_Te_3_ using solvothermal synthesis and estimated their thermoelectric properties^30–32^.

In this study, we fabricated n-type Bi_2_(Se_*x*_Te_1−*x*_)_3_ nanoplates using solvothermal synthesis while significantly changing the atomic composition. The resulting nanoplates were used to form thin films on a flexible substrate using drop-casting. The structural and thermoelectric properties of Bi_2_(Se_*x*_Te_1−*x*_)_3_ nanoplate thin films (NPTs) were evaluated and the optimal atomic composition was determined.

## Methods

Bi_2_(Se_*x*_Te_1−*x*_)_3_ nanoplates were synthesized via a solvothermal process^33^. The outline of this process is described in the supplemental information provided in Fig. S2. We used Bi_2_O_3_ (Fujifilm Wako Pure Chemical Co., >99.9%), TeO_2_ (Kojundo Chemical Laboratory Co., Ltd., >99.9%), SeO_2_ (Fujifilm Wako Pure Chemical Co., >97.0%), ethylene glycol (Fujifilm Wako Pure Chemical Co., >90.0%), polyvinyl pyrrolidone (PVP) (Fujifilm Wako Pure Chemical Co., K30, M_s_ ~40,000), and sodium hydroxide (NaOH) (Fujifilm Wako Pure Chemical Co., >97.0%) to prepare the precursor liquid-solution. The typical procedure for the synthesis of Bi_2_(Se_*x*_Te_1−*x*_)_3_ nanoplates is as follows: 0.4 g PVP was dissolved in ethylene glycol (18 mL), followed by the addition of Bi_2_O_3_ (20 mM), TeO_2_ (5.4–55.2 mM), SeO_2_ (5.4–55.2 mM), and 2 mL of NaOH liquid-solution (0.5 M). Eleven samples of Bi_2_(Se_*x*_Te_1−*x*_)_3_ nanoplates were prepared while the atomic composition of Se was changed from *x* = 0.08 to 0.92. It is to be noted that the pure Bi_2_Te_3_ nanoplates (*x* = 0) and their thin films were prepared in our previous reports, and the structural and thermoelectric properties were discussed^34^. However, it was possible to fabricate the pure Bi_2_Se_3_ nanoplates (*x* = 1.0) using the solvothermal method in our process. The resulting precursor liquid-solutions were sealed in an autoclave, heated, and maintained at 200 °C for 20 h with stirring of the liquid-solution at 500 rpm. After the solvothermal synthesis, the liquid-solutions were naturally cooled to below 50 °C. The nanoplates were then collected via centrifugation and washed several times in distilled water and absolute ethanol. Finally, they were dried in vacuum at 60 °C for 1 day.

The phase purity and crystal structure of Bi_2_(Se_*x*_Te_1−*x*_)_3_ nanoplates were characterized by X-ray diffraction (XRD, Bruker D8 ADVANCE) using Cu-K_α_ radiation (*λ* = 0.154 nm) at a scanning rate of 0.03°/s with 2*θ* ranging from 10° to 80°. The atomic composition of the nanoplates was examined using an electron probe micro analyzer (EPMA, Shimadzu EPMA-1610). The compositions of the samples were calibrated using the installed ZAF4 program of the EPMA-1610. The surface morphology of the Bi_2_(Se_*x*_Te_1-*x*_)_3_ nanoplates was investigated using a field emission scanning electron microscopy (FESEM, Hitachi S-4800).

To measure the thermoelectric properties of the Bi_2_(Se_*x*_Te_1−*x*_)_3_ nanoplates, NPTs were prepared via drop-casting^35,36^. The outline of the drop-casting process is described in the supplemental information provided in Fig. S3. Then, 30 mg of nanoplates were ultrasonically dispersed in 3.0 mL of methanol to form the Bi_2_(Se_*x*_Te_1−*x*_)_3_ nanoplate ink. The nanoplate ink was deposited on a polyimide substrate using a plastic enclosure so that the NPTs were thick with a uniform thickness. The average thickness of NPTs is approximately 4 μm and the standard deviation is 0.3 μm. After drying the NPTs in air, thermal annealing was performed to evaporate the residual matter in the NPTs and to connect the nanoplates to each other, based on our previous study^37^. A furnace was filled with a mixture of argon (95%) and hydrogen (5%) at atmospheric pressure. The gas flow rate was maintained at 1.0 slm throughout the annealing process. The temperature was maintained at 250 °C, and a heating rate of 4 K/min was used; the samples were annealed at the set temperature for 1 h. After thermal annealing, the samples were naturally cooled to below 70 °C. In our previous report, we confirmed that the atomic composition of the nanoplates did not change during thermal annealing at 250 °C^37^.

The in-plane Seebeck coefficient *S* of the Bi_2_(Se_*x*_Te_1-*x*_)_3_ NPTs was measured near 300 K with accuracy of ±5%. One end of the thin film was connected to a heat sink and the other end to a heater. The Seebeck coefficient was determined as the ratio of the potential difference (Δ*V*) along the film to the temperature difference (Δ*T*). The in-plane electrical conductivity *σ* of the NPTs was measured near 300 K using a four-point probe method (Napson, RT-70V) with accuracy of ±3%. The in-plane power factor *σS*^2^ was obtained from the experimentally measured Seebeck coefficient and electrical conductivity. The carrier concentration *n* and mobility *μ* were measured using the van der Pauw’s method (HM-055, Ecopia).

## Results and discussion

**Figure 1:**
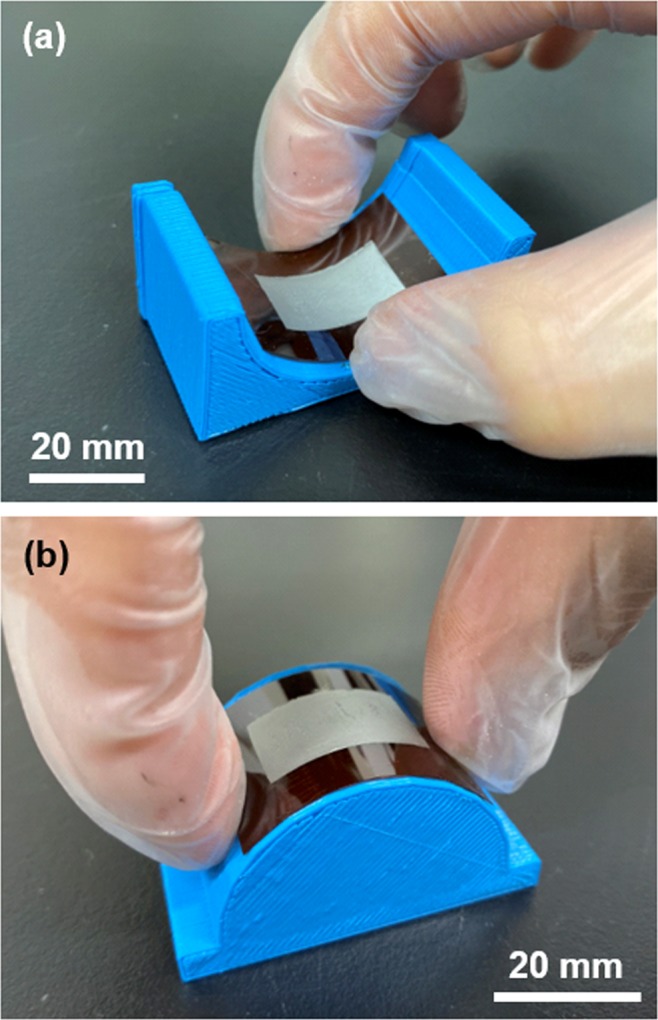
Images of typical Bi_2_(Se_*x*_Te_1−*x*_)_3_ nanoplate thin film (*x* = 0.75) on polyimide substrate for a bending condition. (**a**) Concave shape with a curvature radius of 20 mm, (**b**) convex shape with a curvature radius of 20 mm.

An image of a typical Bi_2_(Se_*x*_Te_1−*x*_)_3_ nanoplate thin film (*x* = 0.75) on a polyimide substrate is shown in Fig. 1. The thin film firmly adhered to the substrate applying compressive stress (Fig. 1(a)) and tensile stress (Fig. 1(b)). To use NPTs in real-world applications, we conducted bending tests by applying compressive stress and tensile stress on the NPTs possessing a high power factor. When the NPTs were repeatedly bent 450 times, the relative variation of resistance was measured. The detailed experimental setup and results are shown in Fig. S4. As a result, the relative resistance after the bending (450 times) was approximately two times higher than that of the initial resistance along both stressing directions. No peeling behavior was observed in the NPTs after the completion of the bending tests.

**Figure 2:**
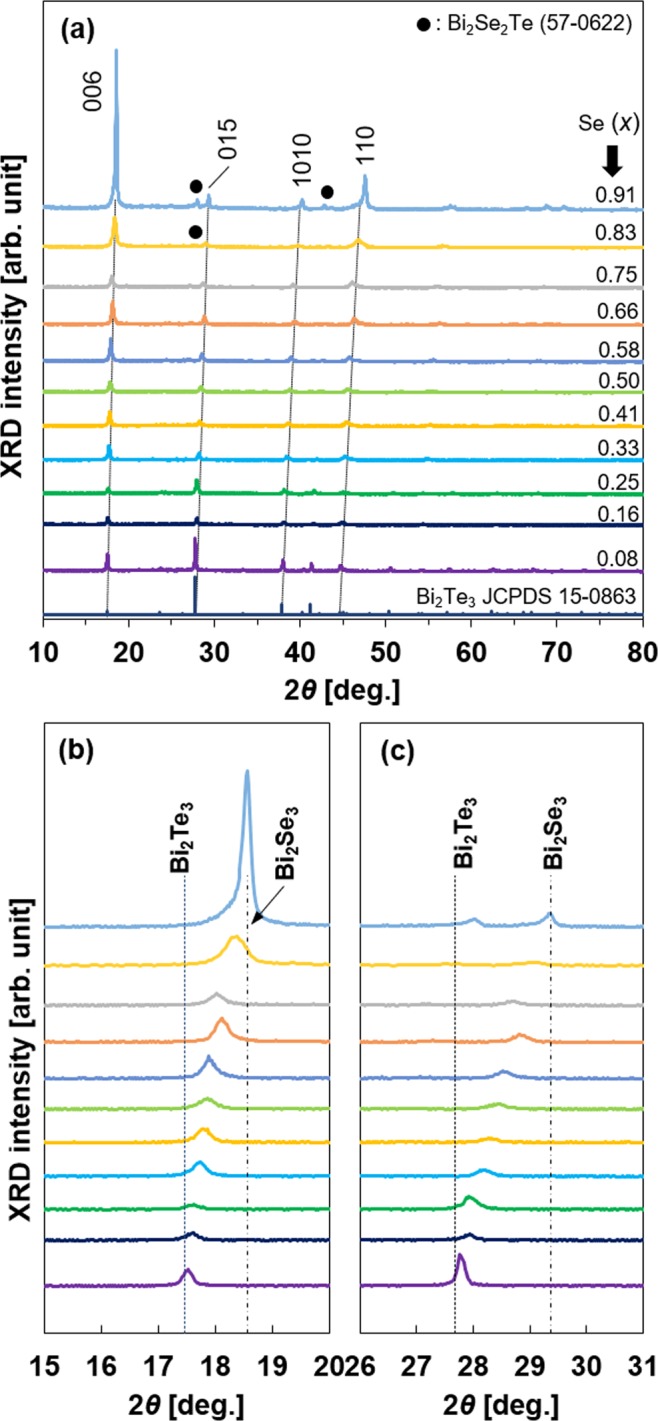
XRD patterns of the Bi_2_(Se_*x*_Te_1−*x*_)_3_ nanoplates obtained at various Se compositions. (**a**) 2*θ* : 10–80°, (**b**) 2*θ* : 15–20°, (**c**) 2*θ* : 26–31°.

X-ray diffraction (XRD) patterns of the Bi_2_(Se_*x*_Te_1-*x*_)_3_ nanoplates with different Se compositions are shown in Fig. 2(a). The diffraction peaks of the Bi_2_(Se_*x*_Te_1-*x*_)_3_ nanoplates (*x* = 0.08) can be closely indexed as the rhombohedral Bi_2_Te_3_ phase (JCPDS No. 15-0863), and no other phases were observed. As the Se composition increased until *x* = 0.75, the detected peaks gradually shifted to the higher angle, and other phases were not observed. This indicates that the Te atoms were replaced by the Se atoms in the unit cell, and the unit cell volume decreased because the atomic radius of Se is smaller than that of Te. Therefore, we conclude that the ternary alloy system of Bi_2_(Se_*x*_Te_1-*x*_)_3_ was successfully synthesized in this composition region. When the Se composition exceeded *x* = 0.83, another phase of Bi_2_Se_2_Te (JCPDS No. 57-0622) with a skippenite crystal structure was observed owing to the disordered occupation of Te/Se atoms in the quintuple layer^38^. The Bi_2_Se_2_Te crystals are usually used as topological insulators^39,40^. To perform a detailed analysis of the crystal structure, the enlarged views of the 006 and 015 peaks are presented in Fig. 2(b,c), respectively. The vertical dashed lines and dashed-dotted lines in the figures represent the peak positions of Bi_2_Te_3_ (JCPDS No. 15-0863) and Bi_2_Se_3_ (JCPDS No. 33-0214), respectively. It is clearly seen that both peaks are shifted from the dashed lines to the dashed-dotted lines with the increase in the Se composition. This indicates that the lattice parameters decreased with increasing Se composition.

**Figure 3:**
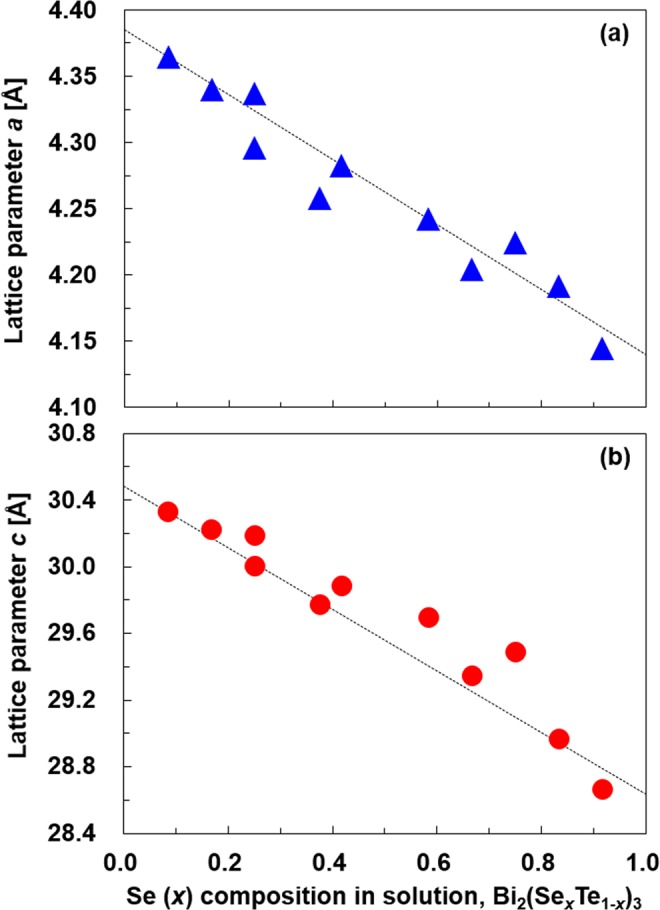
Lattice parameters of Bi_2_(Se_*x*_Te_1−*x*_)_3_ nanoplates for various Se compositions, measured from the XRD peaks. (**a**) *a*-axis and (**b**) *c*-axis.

Based on the results in Fig. 2(b,c), we calculated the lattice parameters *a* and *c*. Figure 3 shows the relationship between the Se composition in Bi_2_(Se_*x*_Te_1-*x*_)_3_ and the lattice parameters. The dashed lines correspond to Vegard’s law. In Fig. 3(a), the lattice parameter *a* is consistent with Vegard’s law over the entire range of Se compositions. However, in Fig. 3(b), the lattice parameter *c* exhibits a positive deviation over the range from Se composition *x* = 0.42 to 0.83. A similar phenomenon was reported in bulk samples by Wiese *et al*.^41^. This observation was explained in terms of Se-atom substitution in preferred planes in the range Bi_2_Te_3_-Bi_2_Te_2_Se. Therefore, the trend shown in Fig. 3(b) occurred not only in the nanoplates (2D material) but also in the bulk samples (3D material).

**Figure 4:**
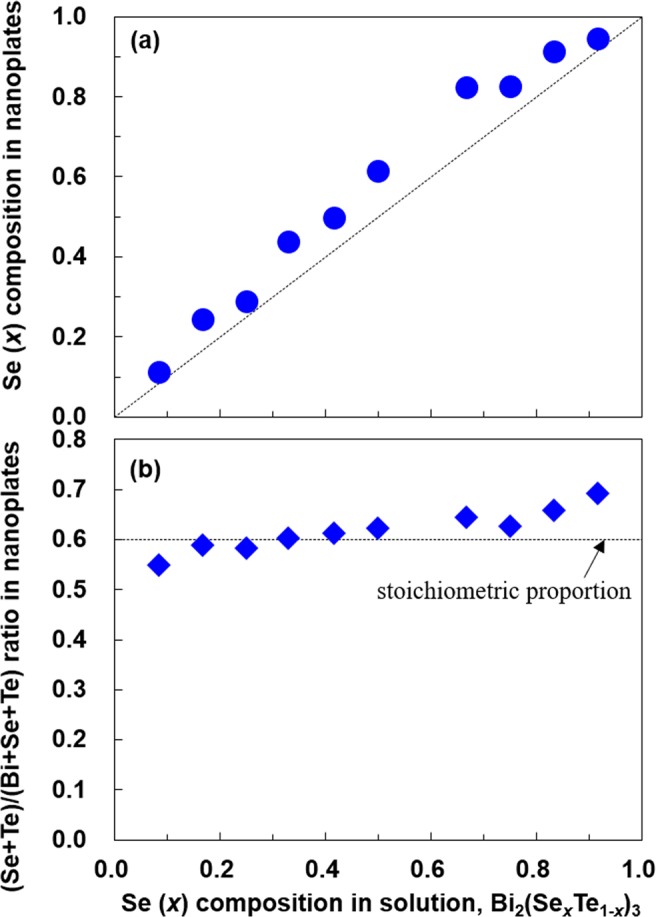
(**a**) Se composition in nanoplates and (**b**) (Se + Te)/(Bi+Se+Te) ratio of Bi_2_(Se_*x*_Te_1−*x*_)_3_ nanoplates as a function of the Se composition in solution, measured using EPMA.

The relationship between the Se composition in the liquid-solution for solvothermal synthesis and the corresponding nanoplates is shown in Fig. 4(a). The diagonal dashed line in this figure represents identical Se composition in both the liquid-solution and the nanoplate. The Se composition in the nanoplates increased as their composition in the liquid-solution was increased along the diagonal line. Therefore, the successful synthesis of a ternary alloy system of Bi_2_(Se_*x*_Te_1-*x*_)_3_ was proved using the EPMA. It should be noted that most of the plots exceeded the diagonal dashed line. This indicates that Se atoms are more easily incorporated into the nanoplate crystals than the Te atoms because the former has a smaller radius and higher electronegativity compared to the latter^26^. In Fig. 4(b), the relationship between the Se composition in the liquid-solution and the (Se + Te)/(Bi+Se+Te) ratio in the corresponding nanoplates is presented. The horizontal dashed line represents the stoichiometric proportion of Bi_2_(Se_*x*_Te_1-*x*_)_3_. The (Se + Te)/(Bi+Se+Te) ratio in the nanoplates exhibited a slightly positive deviation from the stoichiometric proportion when the Se composition in liquid-solution was over *x* = 0.42. At a Se composition of *x* = 0.92, the ratio increased, possibly because another phase of Bi_2_Se_2_Te appeared.

**Figure 5:**
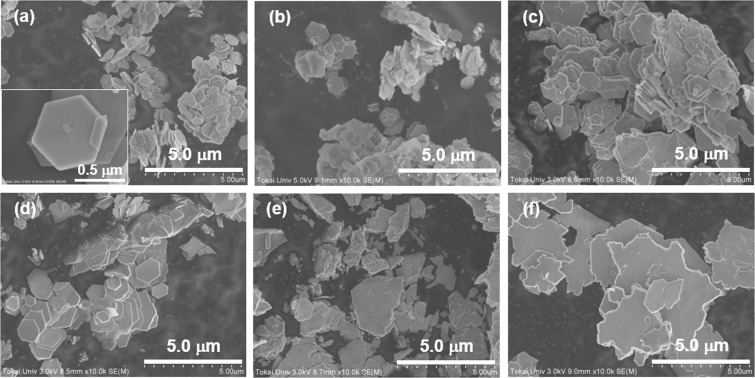
Surface SEM images of the Bi_2_(Se_*x*_Te_1−*x*_)_3_ nanoplates. (**a**), (**b**), (**c**), (**d**), (**e**), and (**f**) corresponding to the Se composition of *x* = 0.08, 0.25, 0.41, 0.58, 0.75, 0.92, respectively.

Figure 5 shows surface SEM images of typical Bi_2_(Se_*x*_Te_1-*x*_)_3_ nanoplates. At a Se composition of *x* = 0.08, high-quality regular-hexagonal nanoplates with diameter of approximately 500 nm and thicknesses less than 50 nm are observed in Fig. 5(a). An enlarged image of the nanoplates is shown in the inset of Fig. 5(a). A screw-dislocation-driven spiral growth appears on the nanoplate’s surface. A similar phenomenon was reported in previous studies of Bi_2_Se_3_ and Sb_2_Te_3_ nanoplates^42,43^. In addition, the radius of critical nucleus *r*_*c*_ can be calculated from the spacing between two neighboring turns *λ*, giving *λ* = 19*r*_*c*_.^44^ In this case, the measured *λ* was 23 nm, so that *r*_*c*_ is determined to be approximately 1.2 nm, which is 2.8 times larger than the lattice parameter *a* in a unit cell with a corresponding Se composition. When the Se composition was increased from *x* = 0.25 to 0.58, the edge size of the nanoplates increased and their shape gradually deviated from regular hexagon (Fig. 5(b–d)). When the Se composition was further increased to *x* = 0.75, the variation of the plate size increased from 0.5 to 3 μm, and the shape of the plates became random (Fig. 5(e)). In addition, when the Se composition had the highest value (*x* = 0.92), the nanoplates gathered to form large plates with a size more than 5 μm (Fig. 5(f)). Thus, although the detailed mechanism of the crystal growth was not elucidated, the experimental results clearly show differences in the shape and size of the nanoplates depending on the Se composition. A possible growth mechanism of the Bi_2_(Se_*x*_Te_1-*x*_)_3_ nanoplates is as follows: when the Se composition is relatively low, the Se atoms smoothly replaced the Te atoms at the Te^(2)^ sites. During this process, the regular hexagonal shape of the nanoplates was sustained. After the Se atoms fully occupied the Te^(2)^ sites, these atoms generally began to fill the Te^(1)^ sites via disordered occupation of Te/Se atoms. As a result, the crystal growth of nanoplates is disordered, and consequently, the shape and size of the nanoplates became random. On further increasing the Se composition, the Bi_2_Se_2_Te phase was also present and the shape was completely different from hexagonal^45^.

**Figure 6:**
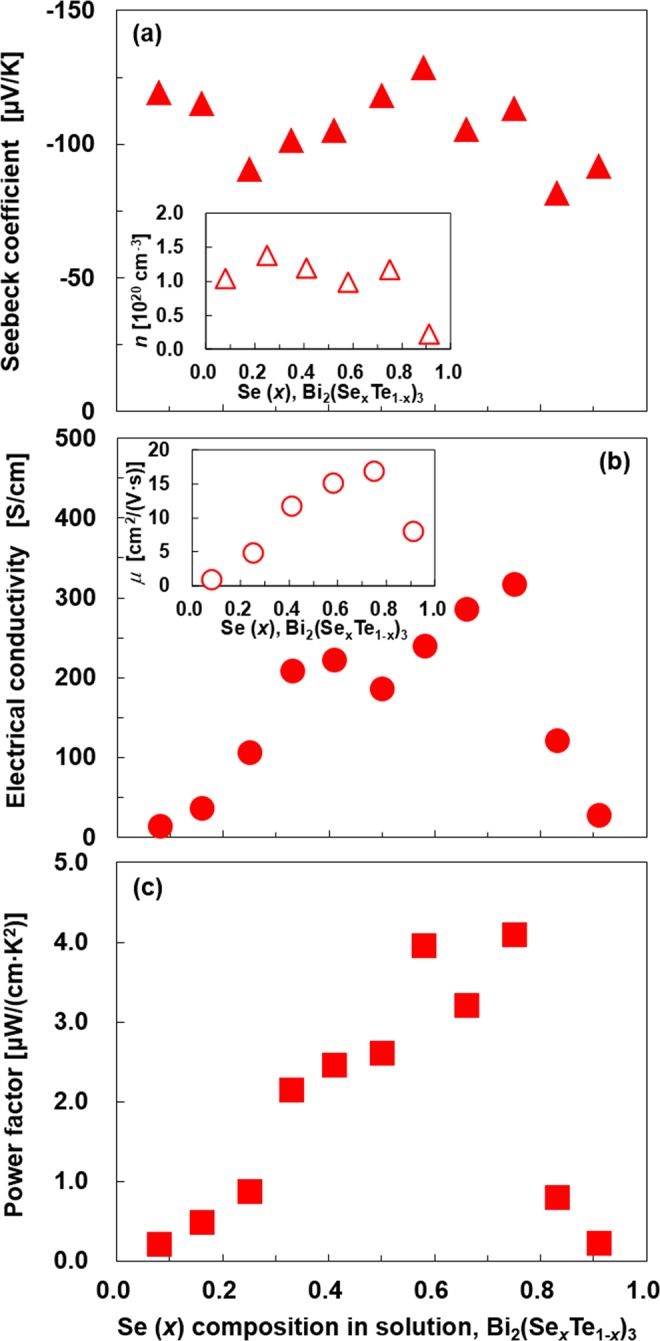
Experimentally measured and calculated thermoelectric properties of the Bi_2_(Se_*x*_Te_1−*x*_)_3_ thin films as a function of Se composition. (**a**) Seebeck coefficients, (**b**) electrical conductivities, and (**c**) power factors. The carrier concentration and mobility as a function of Se composition are shown in the insets of Fig. 6 (**a**) and (**b**), respectively.

Figure 6 shows the relationship between the Se atomic composition and the in-plane thermoelectric properties of the Bi_2_(Se_*x*_Te_1-*x*_)_3_ NPTs on a polyimide substrate. The Seebeck coefficient of the Bi_2_(Se_*x*_Te_1-*x*_)_3_ NPTs as a function of Se composition is shown in Fig. 6(a). For all Se compositions, the samples exhibited negative values. This is indicative of the n-type semiconducting characteristics of the samples. The highest Seebeck coefficient of −128.6 μV/K was obtained for a Se composition of *x* = 0.58. In contrast, the lowest absolute values of −82.0 μV/K was obtained for a Se composition of *x* = 0.83. Thus, a clear relationship between the Se composition and the Seebeck coefficient was not observed. This is because the Seebeck coefficient is strongly related to the carrier concentration, as shown in the inset of Fig. 6(a), which fluctuates with the atomic composition. In addition, defects such as Bi and Te vacancies and antisite defects may also affect the carrier concentration. Especially, the antisite defects are relatively easy to form compared to the vacancies because the formation energy of antisite defects is lower than that of vacancies based on the first-principles studies^46,47^. The NPTs for Se compositions of *x* = 0.83 and 0.92 exhibited lower Seebeck coefficients because of the presence of the Bi_2_Se_2_Te phase^48^.

As shown in Fig. 6(b), the electrical conductivity of the nanoplate thin film at the lowest Se composition (*x* = 0.08) was 15 S/cm. When the Se composition was increased to *x* = 0.75, the electrical conductivity also increased. The highest electrical conductivity of the thin film was 317 S/cm at a Se composition of 0.75. In general, the electrical conductivity of the alloy system tends to decrease as the number of elements and the atomic composition increase because electron scattering is enhanced^28^. However, the experimental results show that the electrical conductivity tends to be higher as the Se composition is increased. This is because the mobility, as shown in the inset of Fig. 6(b), increased due to the increase in the size of the plates. When the Se composition is further increased in excess of *x* = 0.83, the electrical conductivity drastically decreases even though the size of the plates increases. One possible explanation is that the Bi_2_Se_2_Te phase, which is known to achieve large bulk resistivity as a topological insulator^49,50^, disturbed the electron transport.

Figure 6(c) shows the power factor of the Bi_2_(Se_*x*_Te_1-*x*_)_3_ NPTs. The relationship between the power factor and Se composition is similar to that between the electrical conductivity and the Se composition. The power factor increased as the Se composition was increased. At a Se composition of *x* = 0.75, the power factor exhibited the highest value of 4.1 μW/(cm∙K^2^). On further increasing the Se composition, the power factor drastically decreased due to the reduced electrical conductivity.

## Conclusions

In summary, n-type Bi_2_(Se_*x*_Te_1−*x*_)_3_ nanoplates were prepared using solvothermal synthesis while significantly changing the atomic composition. Based on XRD analysis, it was determined that the mixed crystal system of Bi_2_(Se_*x*_Te_1-*x*_)_3_ was successfully synthesized for all Se compositions and these results were supported by analysis using an EPMA. However, when the Se composition was extremely high, the impurity phase appeared. SEM analysis revealed that the shape of the nanoplates changed from a regular hexagon to an irregular shape as the Se composition was increased. The Bi_2_(Se_*x*_Te_1-*x*_)_3_ NPTs were formed on a flexible substrate using drop-casting, followed by thermal annealing. They could not be detached from the substrate even after bending. The electrical conductivity of NPTs increased with the increase in the Se composition but rapidly decreased at a high Se composition. As a result, the Bi_2_(Se_*x*_Te_1-*x*_)_3_ nanoplate thin film exhibited the highest power factor of 4.1 μW/(cm∙K^2^) at a Se composition of *x* = 0.75. We demonstrated that thermoelectric performance can be improved by optimizing the Se composition. The apertures between the NPTs need to be filled with layers composing bismuth-telluride family of compounds using electrodeposition or other deposition methods to well enhance the thermoelectric performance of the NPTs.

## Supplementary information

Supplementary information.
