# Supplementary material for: Solvothermal synthesis of n-type Bi2(SexTe1−x)3 nanoplates for high-performance thermoelectric thin films on flexible substrates
Source: Sci Rep. 2020 Apr 14;10:6315. doi: 10.1038/s41598-020-63374-0 (PMC7156490; doi:10.1038/s41598-020-63374-0)
Supplement: Supplementary file 1 — Supplementary information. [file 41598_2020_63374_MOESM1_ESM.docx]

**Solvothermal synthesis of n-type Bi_2_(Se*_x_*Te_1−_*_x_*)_3_ nanoplates for high-performance thermoelectric thin films on flexible substrates**

Yuki Kimura ^1^, Ryotaro Mori ^1^, Susumu Yonezawa ^1^, Hayato Yabuki ^1^, Hiromasa Namiki ^2^, Yuichi Ota ^2^, Masayuki Takashiri ^1,^*

^1^ *Department of Materials Science, Tokai University, 4-1-1 Kitakaname, Hiratsuka, Kanagawa 259-1292, Japan*

^2^ *Tokyo Metropolitan Industrial Technology Research Institute, 2-4-10, Aomi, Koto-ku, Tokyo 135-0064, Japan*

*Correspondence to takashiri@tokai-u.jp

**Supplemental information**

Figure S1. Crystal structures of (a) Bi_2_Te_3_, (b) Bi_2_Se_3_ binary and (c) Bi_2_(Se*_x_*Te_1-_*_x_*)_3_ ternary alloys.

Figure S3. Outline of the drop-casting process of Bi_2_(Se*_x_*Te_1-_*_x_*)_3_ NPTs.

Figure S2. Outline of the solvothermal synthesis of Bi_2_(Se*_x_*Te_1-_*_x_*)_3_ nanoplates.

Figure S4. Photographs of plastic molds using 3D printer. (a) Concave shape with a curvature radius of 20 mm, (b) convex shape with a curvature radius of 20 mm.

After repeatedly bending the Bi_2_(Se*_x_*Te_1−_*_x_*)_3_ NPTs, which possessed the highest power factor (*x* = 0.75), we analyzed the relative variation of resistance of the NPTs. First, we fabricated two types of plastic molds using a 3D printer (da Vinci1.0 AIO 3D, XZY printing). One type of mold had a concave shape to test the compressive stress and another had a convex shape to test the tensile stress. Both had the same curvature radius of 20 mm. For analysis, the back sides of the samples were pressed against the concave- or convex-shaped molds for 450 times, and the resistance was measured 45 times (10 times for each attempt). Figure S4 shows the relative variation of resistance in the NPTs. When the compressive stress was applied on the film after being bent repeatedly for 100 times, the relative resistance reached 1.60 and slightly increased as the number of bending cycles increased. When the number was 450, the relative resistance was 1.85. When tensile stress was applied to the film, the magnitude of the relative variation of resistance of the film was lower compared to the application of compressive stress, but the trend was similar for both applications. The relative resistance reached 1.41 at 100 and slightly increased as the number of bending cycles increased. At 450, the relative resistance was 1.58. Therefore, compared with the tensile stress, the compressive stress is mainly affected by the varying resistance. We found that there was no peeling behavior in the NPTs after both the bending tests were complete.
